# On the Application of Chloroform in Neuralgia and Muscular Rheumatism

**Published:** 1866-05

**Authors:** Dupuy De Frenelle


### On the Application of Chloroform in Neuralgia and Muscular Rheumatism.

BY DR. DUPUY DE FRENELLE.

Dr. Dupuy maintains that idiopathic neuralgia and muscular rheumatism are two varieties of the same disease, the nerves of common sensation being equally affected in both, and the causes of both being the same. The treatment by chloroform is not new; but Dr. Dupuy contends that no one has previously employed it in the same manner as himself, except the late Dr. Aran. Dr. Dupuy states that he has discovered the means of inducing every variety of local irritation by the contact of chloroform, from simple rubefaction to vesication, the revulsive action being necessary to the success of the plan, which is described as follows: The middle of a piece of fine and well-worn linen is introduced as a stopper into a phial of pure chloroform, which is inverted so as to impregnate with the fluid a more or less considerable portion of the compress, according to the extent of the skin to be acted on; the linen is then laid over the seat of pain, and with the palm of the hand is kept in close contact with the skin; but when the pain is limited to one spot, as in some cases of intercostal, facial, supra-orbital, or auricular neuralgia, the pressure of the thumb or forefinger is sufficient. Several successive applications should be made at once when the pain occupies more than one region, or when it exists along the entire course of a nerve, as in sciatica. In the latter case, the chloroform should be applied over the ischiatic notch, the head of the fibula, or the external malleolus, from the origin of the nerve to its termination. Dr. Dupuy brings forward in support of his practice an hundred and fifty cases, in which the largest number of applications of chloroform was twelve, and this number he has never exceeded.—*British and Foreign Medico-Chirurgical Review, July*, 1864, *p.* 246.

